# Investigation of supplement use and knowledge among Japanese elite athletes for the Tokyo 2020 Olympic/Paralympic games and the Beijing 2022 winter Olympic/Paralympic games

**DOI:** 10.3389/fspor.2023.1258542

**Published:** 2023-10-18

**Authors:** Kanae Myoenzono, Jun Yasuda, Eri Takai, Akiho Shinagawa, Noburo Kaneko, Takahiro Yoshizaki, Keiko Namma-Motonaga, Masae Yoshino, Emi Kondo, Kohei Nakajima, Mika Hangai, Kazuyuki Kamahara, Etsuko Kamihigashi, Shusuke Kusano, Akiko Kamei

**Affiliations:** ^1^ Japan Institute of Sports Sciences, Japan High Performance Sport Center, Tokyo, Japan; ^2^ Department of Health Management, Tokai University, Kanagawa, Japan; ^3^ Department of Food and Life Sciences, Faculty of Food and Nutritional Sciences, Toyo University, Gunma, Japan; ^4^ Department of Nutrition, Faculty of Health and Nutrition, Yamanashi Gakuin University, Yamanashi, Japan; ^5^ Japan Society for the Promotion of Science, Tokyo, Japan; ^6^ Institute of Health and Sport Sciences, University of Tsukuba, Ibaraki, Japan; ^7^ Department of Occupational Therapy, Tokyo Professional University of Health Sciences, Tokyo, Japan

**Keywords:** Olympic athletes, Paralympic athletes, anti-doping, supplement, dietary habits

## Abstract

Elite athletes frequently invest in the use of supplements to optimize their dietary regimens and enhance their athletic performance. However, unregulated and unplanned use of supplements can lead to adverse consequences, including anti-doping rule violations or health issues. Thus, athletes should verify their diets, consider scientific evidence, and take necessary precautions regarding supplements before use. To date, no study has explored whether athletes check these factors before using supplements. This study aimed to investigate supplement use using a questionnaire administered to 1,392 athletes (including candidate athletes) who participated in the Tokyo 2020 Olympic/Paralympic and Beijing 2022 Winter Olympic/Paralympic Games. Participants were categorized as follows: 1,040 participants in the Tokyo 2020 Olympic Games, 83 in the Tokyo 2020 Paralympic Games, 239 in the Beijing 2022 Winter Olympic Games, and 30 in the Beijing 2022 Winter Paralympic Games. We collected data on supplement use and gained further knowledge through interviews with the athletes. Approximately 70% of Tokyo 2020 Olympic/Paralympic and Beijing 2022 Winter Olympic athletes and approximately 50% of Beijing 2022 Winter Paralympians used supplements. Over 50% of athletes had not received a doctor's diagnosis or a dietitian's evaluation before supplement use. Moreover, only 50% of the athletes who used dietary supplements reviewed the scientific evidence for the dietary supplements before using them and justified their choice based on their own investigation, while those who did not use dietary supplements cited either a lack of need or fear of an anti-doping rule violation. Considering the holistic health and performance of athletes, as well as the risk associated with unregulated use, such as overdose and anti-doping rule violations, there is a need for nutritional education on supplement use for athletes and their entourages.

## 1. Introduction

Elite athletes frequently invest in supplements to optimize their diet and enhance their athletic performance. We have been investigating the dietary intake, percentage of supplement use, and purpose of supplement use among Olympic and Paralympic athletes (including candidates) at the Japan Institute of Sport Sciences (JISS) (1–3). Our findings indicated that elite athletes were simultaneously concerned about inadequate dietary intake while aiming for a well-balanced diet. Thus, they turn to supplement use with the expectation of additional benefits, such as enhanced recovery from fatigue and improved athletic performance (1, 3). In particular, a survey conducted during the Rio 2016 Olympic Games (R-OG) confirmed that more than 90% of Japan's national teams made use of supplements (3). Lauritzen and Gjelstad also extracted information from 10,418 Doping Control Forms collected by Anti-Doping Norway between 2015 and 2019 and used supplements among athletes who participated in doping controls. The results of the study report that approximately half of the respondents used supplements (4).

Moreover, the Japanese Parasports Association (JPSA) disclosed that 52 of 140 athletes (37%) who participated in the 8th Far East and South Pacific Games for the Disabled (FESPIC Games) in Busan, South Korea, in 2002 reported using some form of supplement (5). This study is the first to report on supplement use among Japanese Paralympians. Subsequently, it was reported that 84.4% of wheelchair rugby players in Canada use supplements with the primary goal of enhancing athletic performance (6).

In recent years, the International Olympic Committee (IOC) has put forth a supplement consensus statement that recommends that the decision to use supplements should be based on several confirmation items, including a doctor's diagnosis and nutritional assessment by a registered dietitian. The background for this consensus statement stems from prior research findings indicating that supplements have the potential to enhance athletic performance (7) and maintain the health of athletes (8), provided athletes adhere to specific usage conditions. Conversely, there have been documented instances of anti-doping violations attributed to the contamination of doping agents within supplements (9). Consequently, athletes, coaches, and their support teams must carefully weigh these risks and benefits when deciding whether to incorporate supplements into their regimen and when selecting the specific types of supplements. Furthermore, it is crucial to note that anti-doping rule violations can be established even in cases where athletes were neither intentional nor negligent, irrespective of the length of the ensuing sanctions. This principle, commonly referred to as “Strict Liability,” is explicitly outlined in the decisions of numerous sports arbitration tribunals, including the Court of Arbitration for Sport (CAS) in Lausanne, Switzerland (10, 11). Therefore, it is important that athletes possess the needed knowledge regarding the appropriate use of supplements. To the best of our knowledge, no study has investigated and confirmed whether athletes' habitual use of supplements meets the criteria outlined in the IOC consensus statement. Thus, this study aimed to investigate the use of supplements by administering a questionnaire to 1,392 athletes (including candidate athletes) of the Tokyo 2020 Olympic Games (T-OG)/Tokyo 2020 Paralympic Games (T-PG), and Beijing 2022 Winter Olympic Games (B-OG)/ Beijing 2022 Winter Paralympic Games (B-PG). These dietary investigations and surveys of athletes' attitudes toward supplements will provide important information for developing effective nutritional support strategies.

## 2. Methods

### 2.1. Participants

A descriptive study was designed to explore the characteristics of dietary habits and their recognition of supplement use in Japanese athletes using quantitative survey data collected before the Olympic and Paralympic Games. The study participants consisted of all 1,392 Japanese elite athletes who were either Olympic or Paralympic representatives or candidates and had undergone medical checkups at the Japan Institute of Sport Sciences (JISS) (Table 1). The olympic athletes or candidates completed a quantitative survey as part of a medical checkup and the survey data were then confirmed by an interview with a trained dietitian at JISS. Informed consent for Olympic athletes was obtained as an opt-out on the website. Conversely, interviews with Paralympic athletes were conducted following an explanation of the study and after obtaining their informed consent. These interviews for Paralympic athletes took place at either the training camp of each athletic organization or during individual use of the Japan High Performance Sport Center (HPSC), which includes the national training center. In this study, the Tokyo 2020 Olympians and candidate athletes are described as “T-Olympian (*n*  =  1,040),” Tokyo 2020 Paralympian and candidate athletes are described as “T-Paralympian (*n*  =  83),” Beijing 2022 Winter Olympian and candidate athletes are described as “B-Olympian (*n*  =  239),” and Beijing 2022 Winter Paralympian and candidate athletes are described as “B-Paralympian (*n*  =  30).”

**Table 1:** Demographic and dietary status in the Tokyo 2020 games and Beijing 2022 winter games athletes.

		Tokyo 2020 Olympic		Tokyo 2020 Paralympic		Beijing 2022 Olympic		Beijing 2022 Paralympic	
*n*		1,040		83		239		30	
Age, years		26.4	(5.1)	36.1	(9.7)	24.7	(5.3)	35.7	(9.8)
Sex/women, *n*		512	(49.2)	20	(24.1)	110	(46.0)	9	(30.0)
Medication, *n*		30	(2.9)	43	(51.8)	5	(2.1)	14	(46.7)
Nutrition questions									
(1)	Having a staple food, main dish, and side dish ≥twice/day, days/week	5.8	(1.7)	5.1	(2.4)	5.9	(1.7)	4.9	(2.5)
(2)	Having all three meals (breakfast, lunch, and dinner) in a day, days/week	6.2	(1.7)	5.7	(2.2)	6.3	(1.6)	5.6	(2.0)
(3)	Having confectionery or soft drinks (sweetened juice, sweetened canned coffee, etc.)^a^ Excluding sports drinks, days/week	3.3	(2.5)	2.9	(2.5)	2.8	(2.5)	2.9	(2.2)
(4)	Alcohol consumption, days/week	0.5	(1.1)	0.8	(1.7)	0.4	(0.9)	1.0	(2.0)
(5)	Do you know the significance of snacking in sports (function, role, and usage)?/Yes, *n*	937	(90.1)	73	(88.0)	220	(92.1)	28	(93.3)
(6)	Do you take sports foods (sports drinks, jelly, gels, blocks, bars, etc.)?/Yes, *n*	863	(83.0)	77	(92.8)	188	(78.7)	22	(73.3)
(7)	Do you hydrate during your practices or competitions?/Yes, *n*	1,030	(99.0)	83	(100.0)	239	(100.0)	30	(100.0)
(8)	Have you ever had any nutritional support (seminars or counseling)?/Yes, *n*	936	(90.0)	74	(89.2)	215	(90.0)	26	(86.7)
(9)	Do you use any supplements?/Yes, *n*	793	(76.3)	62	(74.7)	176	(73.6)	17	(56.7)
(9)-1	Do you carefully pay attention to anti-doping recommendations when using supplements?/Yes, *n*^a^	761	(98.8)	62	(100.0)	175	(99.4)	17	(100.0)
(9)-2	Ingredients (multiple answers allowed)								
(9)-2-1	Protein powder, *n*	577	(72.8)	51	(82.3)	127	(72.2)	11	(64.7)
(9)-2-2	Amino acids, *n*	523	(66.0)	53	(85.5)	111	(63.1)	13	(76.5)
(9)-2-3	Vitamins, *n*	254	(32.0)	23	(37.1)	76	(43.2)	5	(29.4)
(9)-2-4	Mineral, *n*	140	(17.7)	19	(30.6)	36	(20.5)	5	(29.4)
(9)-2-5	Fatty acids, *n*	64	(8.1)	6	(9.7)	8	(4.5)	3	(17.6)
(9)-2-6	Probiotics, *n*	44	(5.5)	3	(4.8)	5	(2.8)	0	(0.0)
(9)-2-7	Creatine, *n*	96	(12.1)	12	(19.4)	28	(15.9)	1	(5.9)
(9)-2-8	Caffeine, *n*	54	(6.8)	5	(8.1)	13	(7.4)	3	(17.6)
(9)-2-9	β-alanine, *n*	16	(2.0)	2	(3.2)	4	(2.3)	0	(0.0)
(9)-2-10	Sodium bicarbonate, sodium citrate, *n*	4	(0.5)	2	(3.2)	0	(0.0)	2	(11.8)
(9)-2-11	HMB, *n*	26	(3.3)	8	(12.9)	1	(0.6)	1	(5.9)
(9)-2-12	Others, *n*	34	(4.3)	5	(8.1)	11	(6.3)	0	(0.0)

Data are represented as mean (SD) for age and nutrition questions (1)–(4), and number (%) for gender, and nutrition questions (5)–(9) and (9)-1 to (9)-2-12.

^a^ 33 subjects of Tokyo 2020 Olympians did not respond (770 valid responses).

### 2.2. Ethics

The study was reviewed and approved by the Ethics Committee of the JISS (no. 031) and was conducted in accordance with the principles of the Declaration of Helsinki. Information explaining this study to the athletes was presented using the website for T-OG/T-PG (https://www.jpnsport.go.jp/hpsc/Portals/0/resources/jiss/pdf/optout/optout_20210215-2.pdf; accessed on September 19, 2023) and for B-OG/B-PG (https://www.jpnsport.go.jp/hpsc/Portals/0/resources/jiss/pdf/optout/optout_2021_019.pdf; accessed September 19, 2023). Informed consent was obtained from all participants before the administration of the questionnaires and conducting the interviews. For Olympic athletes, as an opt-out, the website had contact details for those who did not consent to the use of their data, and there was a system in place for withdrawal of consent at any time. For Paralympic athletes, the consent process informed them that they could withdraw their consent at any time if they wished.

### 2.3. Measurements

Medical checks for T-Olympians and B-Olympians were conducted 1-5 months before the competition. As part of these evaluations, participants completed either a self-reported questionnaire or a specialized medical checkup original questionnaire application (NOBORI, PSP Corporation, Tokyo, Japan) on dietary habits and supplement use within the year before their medical evaluation. Questionnaires submitted by the participants were checked for missing data and inconsistencies by a well-trained dietitian. Besides the sport, age, and sex, the questionnaire included the following 11 items about dietary habits: (1) Having a staple food, main dish, and side dish ≥twice/day (times/week); (2) Having all three meals (breakfast, lunch, and dinner) in a day (times/week); (3) Having confectionery or soft drinks (sweetened juice, sweetened canned coffee, etc.), excluding sports drinks (times/week); (4) Alcohol consumption (times/week and mL); (5) Do you understand the purpose of using supplementary food in sports (function, role, and usage)?; (6) Do you consume sports food (sports drinks, jelly, gels, blocks, bars, etc.)?; (7) Do you hydrate during practice or competition? (8) Have you ever received nutritional support (seminars or counseling)? (9) Do you take any supplements?; (9-1) Do you pay careful attention to anti-doping rule violations when using supplements?; (9-2) Type of supplements used?; (10) Confirmation items in accordance with the Supplement Consensus Statement proposed by the IOC on the use of supplements; and (11) Reasons for non-use of supplements (see Appendix 1). Dietitians checked for missing data and rechecked with the athletes if they were questionable. In addition, “supplements” in this study were defined primarily as those in the form of powders, tablets, and capsules (as described in the Appendix), while those in the form of drinks, jellies, blocks and bars were defined as “sports foods”.

### 2.4. Statistics

Microsoft Excel was used for descriptive statistics of all parameters. Data are presented as Mean ± standard deviation (SD) for continuous variables and number (%) for categorical variables.

## 3. Results

### 3.1. Dietary habits and food knowledge for sports in T-OG/T-PG and B-OG/B-PG athletes

The sports organizations in this investigation included 35 sports in the T-OG, 9 in the T-PG, 5 in the B-OG, and 2 in the B-PG (Supplementary Material Table S1). In Supplementary Material Table S1, in both the T-OG/PG and B-OG/PG, the average age of the Paralympic athletes was approximately 10 years older than that of the Olympic athletes (T-Olympian:26.4 ± 5.1 years, T-Paralympian:36.1 ± 9.7 years, B-Olympian:24.7 ± 5.3 years, B-Paralympian:35.7 ± 9.8 years). On average, athletes in the T-OG, T-PG, B-OG, and B-PG consumed staple food, main dish, and side dish at least twice a day for approximately 5.8 days/week, 5.1 days/week, 5.9 days/week, and 4.9 days/week, respectively, in Table 1. Furthermore, the number of athletes in the T-OG, T-PG, B-OG, and B-PG who reported eating such a diet less than four days a week was 155 (14.9%), 39 (47.0%), 24 (10.0%) and 12 (40.0%), respectively. The average number of days athletes in the T-OG, T-PG, B-OG, and B-PG consumed all three meals in a day were 6.2 days/week, 5.7 days/week, 6.3 days/week, and 5.6 days/week, respectively. In T-OG, T-PG, B-OG and B-PG, 90 (8.7%), 19 (22.9%), 16 (6.7%), and 7 (23.3%) athletes respectively reported eating three meals a day less than four days a week. The average number of days that T-OG, T-PG, B-OG and B-PG athletes drank soft drinks (e.g., sweetened juice, sweetened canned coffee) in a day were 3.3 days/week, 2.9 days/week, 2.8 days/week, and 2.9 days/week, respectively. Moreover, the average number of days athletes in the T-OG, T-PG, B-OG, and B-PG consumed alcohol in a week were 0.5 days/week, 0.8 days/week, 0.4 days/week, and 1.0 days/week, respectively. The number of athletes in the T-OG, T-PG, B-OG, and B-PG used sports foods (sports drinks, jelly, gels, blocks, bars, etc.) were 83.0%, 92.8%, 78.7%, and 73.3%, respectively. Almost all participants were hydrated during practices and/or competitions. Approximately 90% of the participants received nutritional support (seminars or counseling). Furthermore, the prevalence of supplement use in the last year among athletes was found to be 76.3%, 74.7%, 73.6%, and 56.7% in the T-OG, T-PG, B-OG, and B-PG, respectively. Most participants paid careful attention to the anti-doping recommendations when and while using supplements. Protein powder and amino acids were the most commonly used supplements among the athletes (Protein powder/ T-Olympian: 72.8%, T-Paralympian: 82.3%, B-Olympian: 72.2%, B-Paralympian: 64.7%; Amino acids/ T-Olympian: 66.0%, T-Paralympian: 85.5%, B-Olympian: 63.1%, B-Paralympian: 76.5%), followed by vitamins and minerals, in both the T-OG/PG and B-OG/PG. The creatine usage was observed among 10%−20% of T-OG and B-OG/PG athletes.

### 3.2. Purpose of supplement use

The purposes of using supplements, as per the participants, are summarized in Table 2. “Recovery” was the most frequent answer among participants, both in the T-OG/PG and B-PG. However, “performance enhancement” was the most frequent answer from participants of B-OG. While many athletes use supplements with clear objectives, others use supplements without any objectives, such as “Recommended by staff (coach, teammate, someone from the product company, etc.)”, “Teammates or other players use it”, “It was free (from product company, team, cafeteria, etc.,)” or “Just in case”.

**Table 2:** Reasons for supplement use in the Tokyo 2020 games and Beijing 2022 winter games athletes.

Reasons	Tokyo 2020 Olympic (*n* = 793)						Tokyo 2020 Paralympic (*n* = 62)						Beijing 2022 Olympic (*n* = 176)						Beijing 2022 Paralympic (*n* = 17)					
	All		Men		Women		All		Men		Women		All		Men		Women		All		Men		Women	
	***n***	**(** **%)**	***n***	**(** **%)**	***n***	**(** **%)**	***n***	**(** **%)**	***n***	**(** **%)**	***n***	**(** **%)**	***n***	**(** **%)**	***n***	**(** **%)**	***n***	**(** **%)**	***n***	**(** **%)**	***n***	**(** **%)**	***n***	**(** **%)**
For weight gain or weight loss	221	(27.9)	127	(30.6)	94	(24.9)	19	(30.6)	13	(26.0)	6	(50.0)	60	(34.1)	42	(43.8)	18	(22.5)	5	(29.4)	5	(38.5)	0	(0.0)
For recovery	405	(51.1)	216	(52.0)	189	(50.0)	36	(58.1)	28	(56.0)	8	(66.7)	81	(46.0)	41	(42.7)	40	(50.0)	9	(52.9)	6	(46.2)	3	(75.0)
For performance enhancement	349	(44.0)	189	(45.5)	160	(42.3)	26	(41.9)	23	(46.0)	3	(25.0)	83	(47.2)	43	(44.8)	40	(50.0)	3	(17.6)	3	(23.1)	0	(0.0)
For energy or nutrients supplementation	229	(28.9)	117	(28.2)	112	(29.6)	22	(35.5)	18	(36.0)	4	(33.3)	62	(35.2)	28	(29.2)	34	(42.5)	7	(41.2)	5	(38.5)	2	(50.0)
To treat or prevent disease and injury or strengthen immune system	54	(6.8)	30	(7.2)	24	(6.3)	8	(12.9)	6	(12.0)	2	(16.7)	8	(4.5)	4	(4.2)	4	(5.0)	1	(5.9)	1	(7.7)	0	(0.0)
For improved sleep quality	8	(1.0)	5	(1.2)	3	(0.8)	3	(4.8)	3	(6.0)	0	(0.0)	2	(1.1)	2	(2.1)	0	(0.0)	0	(0.0)	0	(0.0)	0	(0.0)
For improved intestinal environment	35	(4.4)	18	(4.3)	17	(4.5)	2	(3.2)	2	(4.0)	0	(0.0)	3	(1.7)	1	(1.0)	2	(2.5)	0	(0.0)	0	(0.0)	0	(0.0)
Recommended by staff (coach, teammate, someone from the product company, etc.)	14	(1.8)	4	(1.0)	10	(2.6)	3	(4.8)	3	(6.0)	0	(0.0)	1	(0.6)	1	(1.0)	0	(0.0)	0	(0.0)	0	(0.0)	0	(0.0)
Teammates or other players use it	4	(0.5)	1	(0.2)	3	(0.8)	0	(0.0)	0	(0.0)	0	(0.0)	0	(0.0)	0	(0.0)	0	(0.0)	0	(0.0)	0	(0.0)	0	(0.0)
It was free (from product company, team, cafeteria, etc.,)	12	(1.5)	6	(1.4)	6	(1.6)	1	(1.6)	1	(2.0)	0	(0.0)	1	(0.6)	1	(1.0)	0	(0.0)	2	(11.8)	1	(7.7)	1	(25.0)
It is convenient to supplement energy or nutrients before and/or after exercise	33	(4.2)	9	(2.2)	24	(6.3)	6	(9.7)	5	(10.0)	1	(8.3)	9	(5.1)	5	(5.2)	4	(5.0)	2	(11.8)	2	(15.4)	0	(0.0)
Just in case	8	(1.0)	5	(1.2)	3	(0.8)	3	(4.8)	2	(4.0)	1	(8.3)	0	(0.0)	0	(0.0)	0	(0.0)	0	(0.0)	0	(0.0)	0	(0.0)
Others	10	(1.3)	4	(1.0)	6	(1.6)	2	(3.2)	2	(4.0)	0	(0.0)	3	(1.7)	2	(2.1)	1	(1.3)	3	(17.6)	3	(23.1)	0	(0.0)

Data are represented as number (%).

### 3.3. Confirmation states on supplement use

Table 3 presents the results to guide informed decision-making and reduce the risk of anti-doping rule violations for supplement use. Most athletes used supplements without undergoing professional assessments to determine potential nutrient excess or deficiency (66.2% of T-Olympians, 72.6% of T-Paralympians, 61.4% of B-Olympians, and 64.7% of B-Paralympians). The 8%−18% of participants except B-Paralympian answered “No” to “Considered whether the targeted nutrients cannot be obtained from dietary foods (due to food allergy or training abroad).” Furthermore, it was found that approximately 3%−6% of participants, excluding B-Paralympians, did not verify the efficacy of the supplements that they used. In addition, 11.8%−29.0% of the participants in each game category did not confirm scientific evidence to support their performance-enhancing claims of their supplements. The study revealed that approximately half of the participants used supplements without first confirming potential side effects or interactions between the supplements and their prescribed medications. Furthermore, approximately 5%−10% of the participants, excluding the B-PG, used supplements without ensuring that they did not contain any substances prohibited by WADA.

**Table 3:** Confirmation items for the supplement use in the Tokyo 2020 games and Beijing 2022 winter games athletes.

	Tokyo 2020 Olympic (*n* = 793)						Tokyo 2020 Paralympic (*n* = 62)						Beijing 2022 Olympic (*n* = 176)						Beijing 2022 Paralympic (*n* = 17)					
	Yes		No		Not this purpose		Yes		No		Not this purpose		Yes		No		Not this purpose		Yes		No		Not this purpose	
	*n*	(%)	*n*	(%)	*n*	(%)	*n*	(%)	*n*	(%)	*n*	(%)	*n*	(%)	*n*	(%)	*n*	(%)	*n*	(%)	*n*	(%)	*n*	(%)
(10)-1 Before using the supplements, did you get diagnosed by a doctor or assessed by a dietitian?	268	(33.8)	525	(66.2)	–	–	17	(27.4)	45	(72.6)	–	–	68	(38.6)	108	(61.4)	–	–	6	(35.3)	11	(64.7)	–	–
(10)-1-1 The doctor diagnosed undernutrition	54	(20.1)	214	(79.9)	–	–	2	(11.8)	15	(88.2)	–	–	20	(29.4)	48	(70.6)	–	–	2	(33.3)	4	(66.7)	–	–
(10)-1-2 The dietitian assessed undernutrition using dietary records	92	(34.3)	176	(65.7)	–	–	10	(58.8)	7	(41.2)	–	–	28	(41.2)	40	(58.8)	–	–	2	(33.3)	4	(66.7)	–	–
(10)-2 Considered whether the targeted nutrients cannot be obtained from dietary foods (due to food allergy or training abroad)^a^	440	(55.5)	81	(10.2)	271	(34.2)	35	(56.5)	11	(17.7)	16	(25.8)	126	(71.6)	14	(8.0)	36	(20.5)	12	(70.6)	0	(0)	5	(29.4)
(10)-3 Confirmed that the supplements improve the targeted nutrient deficiency^a^	563	(71.0)	46	(5.8)	183	(23.1)	54	(87.1)	3	(4.8)	5	(8.1)	139	(79.0)	5	(2.8)	32	(18.2)	13	(76.5)	0	(0)	4	(23.5)
(10)-4 Confirmed scientific evidence of enhanced performance^a^	498	(62.8)	160	(20.2)	134	(16.9)	35	(56.5)	18	(29.0)	9	(14.5)	112	(63.6)	34	(19.3)	30	(17.0)	10	(58.8)	2	(11.8)	5	(29.4)
(10)-5 Confirmed side effects or interactions between supplements and medications^a^	391	(49.3)	399	(50.3)	–	–	22	(35.5)	40	(64.5)	–	–	91	(51.7)	85	(48.3)	–	–	8	(47.1)	9	(52.9)	–	–
(10)-6 Confirmation that prohibited substances as defined by WADA are not contained	738	(93.1)	53	(6.7)	–	–	59	(95.2)	3	(4.8)	–	–	159	(90.3)	17	(9.7)	–	–	17	(100)	0	(0)	–	–

Data are represented as number (%). Only subjects who responded that they take supplements were interviewed.

^a^ One subject did not respond to questions ⑩-2 to ⑩-4, and 3 subjects did not respond to questions ⑩-5 of the Tokyo 2020 Olympic athletes.

### 3.4. The sources of information for the supplement use

**Figure 1:**
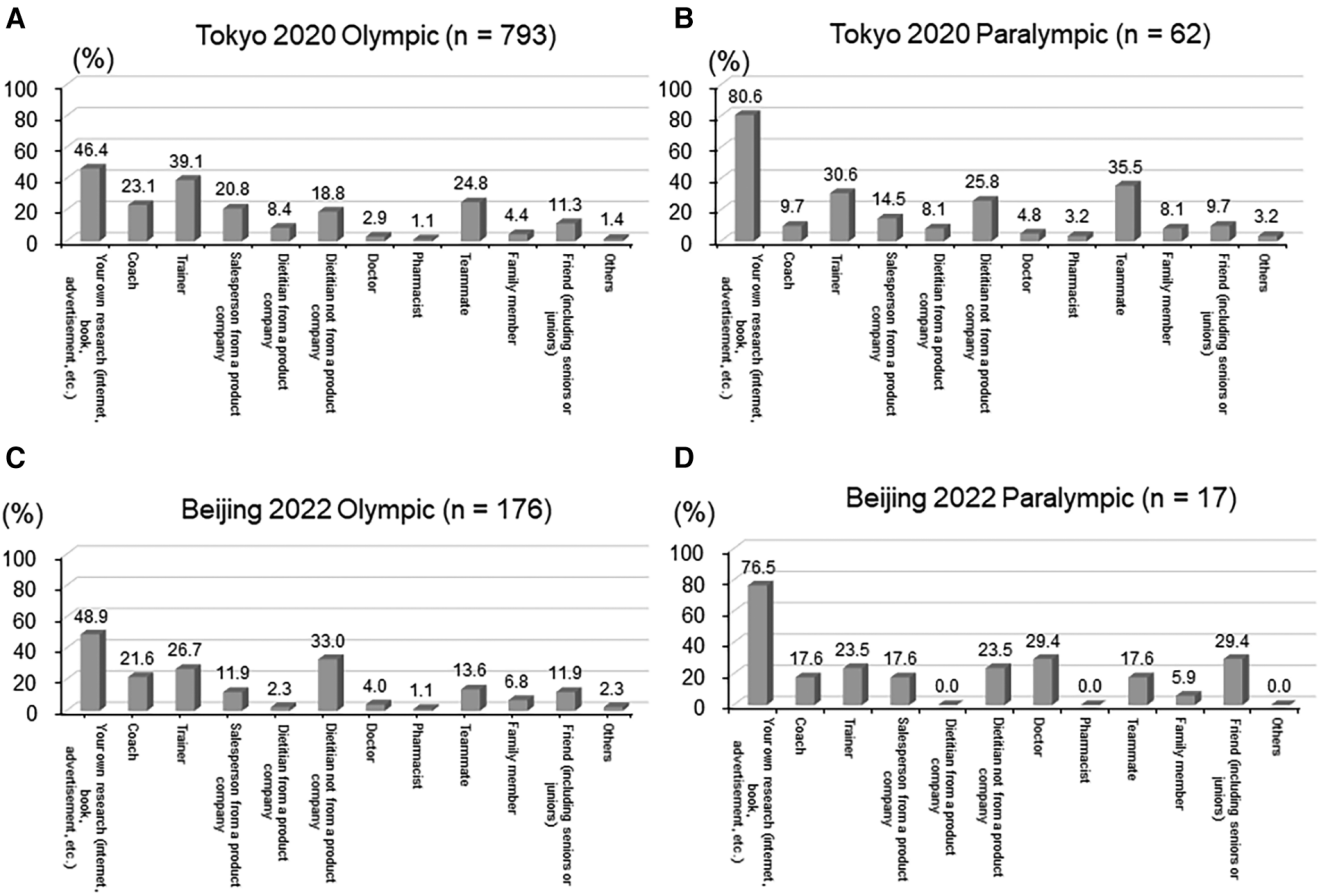
Sources of information that led to the use of the supplement at the T-OG(**A**)/PG(**B**) and the B-OG(**C**)/PG(**D**)..

Figure 1 shows the sources of information that encourage supplemental use. The largest number of participants, both T-OG/PG and B-OG/PG, decided to research the effects and take them on their own (46.4% of T-Olympian (A), 80.6% of T-Paralympian (B), 48.9% of B-Olympian (C), and 76.5% of B-Paralympian (D)). The next most common sources were “Trainers” for T-Olympian (39.1%), “Teammates” for T-Paralympian (35.5%), “Dietitian not from a product company” for B-Olympian (33.0%), and “Doctors”/“Friends (including senior and junior)” for B-Paralympian (29.4%).

### 3.5. Reasons for non-use of supplements

**Figure 2:**
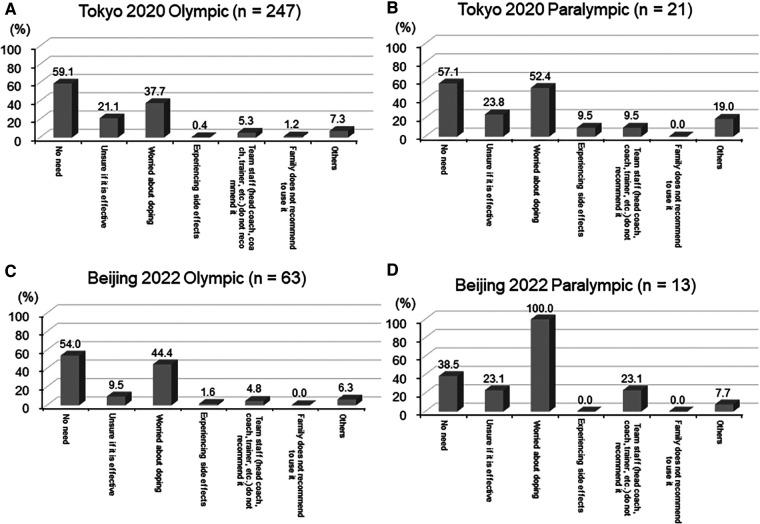
Reasons for non-use of supplements at the T-OG(**A**)/PG(**B**) and the B-OG(**C**)/PG(**D**).

Figure 2 shows the reasons for the non-use of supplements among the participants who did not use supplements. The most common responses were “No need” (59.1% of T-Olympian (A), 57.1% of T-Paralympian (B), 54.0% of B-Olympian (C), and 38.5% of B-Paralympian (D)); and “Concerned about doping” (37.7% of T-Olympian, 52.4% of T-Paralympian, 44.4% of B-Olympian, and 100% of B-Paralympian).

## 4. Discussion

This study investigated supplement use in T-OG/T-PG and B-OG/B-PG athletes using a questionnaire. The results revealed that most elite athletes were concerned about eating a well-balanced diet, hydration during practice, and consuming alcohol and snacks. Conversely, it was discovered that although many elite athletes used supplements, many did not check the decision flowchart outlined by the IOC recommendations, which provides athletes with crucial questions to consider to help them make informed decisions regarding supplement use (12). These results indicate the importance of developing nutritional education for all national team athletes to acquire correct knowledge about diet and supplements and to judge the necessity of supplement use at their own risk.

As shown in Table 1, among the athletes representing both T-OG/PG and B-OG/PG, Paralympic athletes appeared to have a relatively lower dietary quality (unbalanced diet, lower frequency of meals, higher frequency of alcohol consumption) than Olympic athletes, which may be due to the unique background of Paralympic athletes. Individuals with disabilities (e.g., visual impairments, amputation, spinal cord injuries, and cerebral palsy) may find it difficult to perform a series of tasks related to meals by themselves, such as procuring, cooking, and swallowing food. In addition to the difficulty in preparing and consuming food, the energy requirements of Paralympic athletes may influence dietary quality. For example, athletes with spinal cord injuries show reduced energy expenditure during rest and exercise owing to a lower amount of active muscle mass (13). Therefore, nutritional education for Paralympic athletes should be divided according to cases of disability. Several consensus statements and positions in the sports field emphasize that athletes should think about foods first for their health and performance (14–18). Furthermore, considering that neither Olympic nor Paralympic athletes in this study maintained a balanced diet and frequently skipped meals every day, it underscores the need to prioritize nutritional education that emphasizes the importance of maintaining a balanced diet and regular meal consumption. However, since an athlete's diet may also be related to practice schedules and competition characteristics, the importance of providing nutritional support that considers these factors should also be considered. Moreover, Paralympic athletes consumed more alcohol than Olympic athletes in the present study. This may be related to the higher age and ratio of male athletes among Paralympic athletes than among Olympic athletes. Many studies have confirmed that men and older individuals consume more alcohol than women and younger individuals (19, 20). Considering the negative effects of alcohol on physical performance (21) and muscle anabolism (22, 23), education on alcohol consumption may be needed to further improve performance, especially in Paralympic athletes.

To reduce the risk of unintentional doping and prevent nutrient overdoses, the IOC recommends checking a flowchart when using supplements (12). The first confirmation item should be performed by a doctor or sports dietitian. Unfortunately, more than half of the athletes used supplements without a physician's diagnosis or evaluation by a sports dietitian. The results revealed that 106 (7.6%) of the 1,392 athletes in this study used supplements without considering dietary improvements. In addition, as shown in Table 2, some athletes used supplements without checking the efficacy or scientific evidence of nutrient deficiencies and ergogenic aid in this study, even though the purpose of use is clear. Under these conditions, athletes are at an increased risk of overdosing on nutrients, for which there is an acceptable upper limit in Japan (24) and anti-doping rule violations risk. It should be emphasized that most previous studies have examined the effects of supplements after controlling or assessing dietary status (25–30). Moreover, Wickham et al. review the physiological considerations that contribute to the ergogenic effects of supplements (first, the impact of first pass metabolism; second, rises in systemic concentrations; and third, interactions with the target tissue) (31). As can be seen from this review, there is considerable individual variability in the ergogenic effects of supplements, which can be influenced by a variety of factors, including gender, race, body size, timing of supplement intake, amount taken, interactions with other supplements, and metabolic function in the body. Therefore, it is also a very important skill for athletes and their support staff to be able to properly interpret research articles on the effects of supplements. The athletes and their support staff should remember that a handful of supplements can have a small but significant ergogenic effect on their own, such as caffeine. Based on such various backgrounds, our research group summarizes how the “meal first” strategy and planned supplement use are important for enhancing athletes' health and performance, as supplement use by athletes is becoming more common (32). Furthermore, athletes should understand that there is no evidence that they will benefit from supplements alone. Otherwise, the expectation of absolute insurance by supplements leads to the notion that “Using supplements will give you an advantage.” This may undermine the fairness of the sports. To protect the mental and physical health of athletes, it is necessary to thoroughly educate the surrounding environment, including coaches and trainers, on the use of supplements. We also believe that improving athletes’ supplementary literacy is an important strategy to enhance justice, fairness, and integrity in sports, which WADA also emphasizes.

We also found that approximately half of the representative athletes in both the T-OG/T-PG and B-OG/B-PG groups did not check for the side effects of supplements or their interaction with other medications. Excessive caffeine consumption has been reported to worsen sleep and amplify symptoms such as nausea, anxiety, and insomnia (33). In addition, it is crucial to exercise caution and thoroughly check the potential short- and long-term effects of excessive intake of vitamins (particularly fat-soluble vitamins) and minerals, especially when combined with other medications, as the actual effects and the possibility of serious side effects are debated not only among the general population but also within the athletic community. Since a significant number of Olympic athletes (T-Olympian, 97.1%; B-Olympian, 97.9%) in our study had no daily medication with respect to the entire target population, there may have been no need to check for side effects or potential interactions with medications. The survey also revealed that the rate of supplement use was lower among Paralympic athletes than among Olympic athletes. In Japan, the JPSA Anti-Doping Committee conducts regular supplement use and medication surveys as part of athlete medical examinations before the World Championships, Asian Championships and Paralympic Games. Consequently, athletes are reminded to carefully consider the use of supplements at their own risk, taking into consideration the potential interactions between supplements and their regular medications, as well as the risk of developing other diseases due to supplement usage (5, 34). A regular drug survey was also conducted in conjunction with a supplemental use survey (34). Owing to the efforts of the JPSA Anti-Doping Committee, few para-athletes use supplements.

Many studies have confirmed the side effects of using supplements (especially overdoses) (7, 33, 35–39). Geyer and colleagues found that of 634 non-hormonal supplements purchased in 13 countries in 2000–2001, 15% were contaminated with anabolic-androgenic steroids not declared on the label (40). Moreover, in a 2010 review of protein supplements, ConsumerLab reported that tests carried out on 24 commercially available protein supplements found that 31% of the products tested failed their quality assurance test (41). In a more recent review, Martínez-Sanz et al. (42) reported rates of contamination of WADA prohibited substances in ergo-nutritional supplements of between 12% and 58% (42). Kozhuharov et al., concluded that athletes and their teams should be aware of the problems associated with the use of supplements since the analysis of “non-banned” supplements revealed that more than 28% of the supplements analyzed posed a potential risk of unintentional doping (43). Supplements are not required to label all ingredients. Therefore, it is important to note that supplements may contain doping-prohibited substances, and safety cannot be assured solely by ingredient labeling. Even though only 2% of the athletes in this survey were unaware of anti-doping measures when using supplements [Table 1: (9)−1], 5% of the athletes did not check whether the supplements they used contained WADA-doping-prohibited substances [Table 3: (10)−6]. Although athletes might have been aware of anti-doping measures, some athletes might not personally check for prohibited substances. The results suggest that there is a lower tendency for anti-doping awareness of supplements not provided by oneself, such as those supplied or purchased by a team. It was also revealed that the most common information sources for using supplements were themselves, as well as their supervisors/coaches, trainers, and supplement company representatives. When using supplements, it is crucial to choose products that bear the certification batch from reputable third-party certification programs, that take responsibility for the administration of supplements, and consider the safety of the supplements consumed. Those involved in sports should be educated to avoid violating anti-doping rules.

This study had several limitations. Firstly, the survey results on Paralympians were limited to eight sports organizations for the T-PG and two sports organizations for the B-PG; therefore, further study is needed before the results can be generalized. Secondly, the data include players who are based overseas; thus, some athletes may follow a foreign diet for extended periods of time. Thirdly, our results extend not only to Olympians/Paralympians but also to candidates, owing to the inclusion of medical checks in the JISS system. Therefore, it is important to exercise caution when interpreting the results of this study. Lastly, the term dietary supplements encompasses a very heterogeneous group of products with significant differences in content, motivation for use, and health and doping risk. To address these limitations, further studies are needed to clarify the types of supplements in detail.

In conclusion, this study revealed that many athletes were committed to maintaining good eating habits, ensuring proper hydration during sports, and incorporating the consumption of sports foods into their regimen. Furthermore, it was observed that approximately 75% of the participants were using supplements. The use of supplements was lower among Paralympic athletes than Olympic athletes. Additionally, it was also indicated that several athletes used supplements without confirming the quality of their diets and the ingredients present in the supplements. In this study, we were able to gain insights into the dietary characteristics, supplement usage patterns, and awareness of anti-doping measures among Olympic and Paralympic athletes in various sports. Based on the findings of this research, it is imperative to implement tailored nutritional support and anti-doping education specific to the characteristics of each sport. Furthermore, there is a need to update and disseminate information regarding the necessity, effectiveness, and safety of supplements, thereby contributing to the prevention of unintentional anti-doping rule violations among athletes.

## Data Availability

The raw data supporting the conclusions of this article will be made available by the authors, without undue reservation.
