# Corrigendum: Investigation of supplement use and knowledge among Japanese elite athletes for the Tokyo 2020 Olympic/Paralympic Games and the Beijing 2022 Winter Olympic/Paralympic Games

**DOI:** 10.3389/fspor.2023.1338039

**Published:** 2023-11-29

**Authors:** Kanae Myoenzono, Jun Yasuda, Eri Takai, Akiho Shinagawa, Noburo Kaneko, Takahiro Yoshizaki, Keiko Namma-Motonaga, Masae Yoshino, Emi Kondo, Kohei Nakajima, Mika Hangai, Kazuyuki Kamahara, Etsuko Kamihigashi, Shusuke Kusano, Akiko Kamei

**Affiliations:** ^1^ Japan Institute of Sports Sciences, Japan High Performance Sport Center, Tokyo, Japan; ^2^ Department of Health Management, Tokai University, Kanagawa, Japan; ^3^ Department of Food and Life Sciences, Faculty of Food and Nutritional Sciences, Toyo University, Gunma, Japan; ^4^ Department of Nutrition, Faculty of Health and Nutrition, Yamanashi Gakuin University, Yamanashi, Japan; ^5^ Japan Society for the Promotion of Science, Tokyo, Japan; ^6^ Institute of Health and Sport Sciences, University of Tsukuba, Ibaraki, Japan; ^7^ Department of Occupational Therapy, Tokyo Professional University of Health Sciences, Tokyo, Japan

**Keywords:** Olympic athletes, paralympic athletes, anti-doping, supplement, dietary habits

A Corrigendum on Investigation of supplement use and knowledge among Japanese elite athletes for the Tokyo 2020 Olympic/Paralympic games and the Beijing 2022 winter Olympic/Paralympic games By Myoenzono K, Yasuda J, Takai E, Shinagawa A, Kaneko N, Yoshizaki T, Namma-Motonaga K, Yoshino M, Kondo E, Nakajima K, Hangai M, Kamahara K, Kamihigashi E, Kusano S and Kamei A (2023). Front. Sports Act. Living 5:1258542. doi: 10.3389/fspor.2023.1258542

In the published article, references 24 and 39 were incorrectly written. The refences were written as:

“24. Nutrition, N. I. O. H. A. The dietary reference intakes.”

“39. World Anti-Doping Agency. (2020). Anti-doping rule violations (ADRVs) report.”

and should read:

“24. Ministry of Health. Dietary Reference Intakes for Japanese. Available: https://www.mhlw.go.jp/content/001151422.pdf [Accessed 7 Oct 2023]”

“39. World Anti-Doping Agency. (2020). Anti-doping rule violations (ADRVs) report. Available: https://www.wada-ama.org/en/resources/anti-doping-stats/anti-doping-rule-violations-adrvs-report#resource-download [Accessed 7 Oct 2023]”

The authors apologize for these errors and state that this does not change the scientific conclusions of the article in any way. The original article has been updated.

