# Supplementary material for: Investigation of supplement use and knowledge among Japanese elite athletes for the Tokyo 2020 Olympic/Paralympic games and the Beijing 2022 winter Olympic/Paralympic games
Source: Front Sports Act Living. 2023 Oct 18;5:1258542. doi: 10.3389/fspor.2023.1258542 (PMC10622793; doi:10.3389/fspor.2023.1258542)
Supplement: Supplementary file 1 [file Table1.docx]

| Supplementary Table 1．Sports of the Tokyo 2020 Games and Beijing 2022 Winter Games Athletes. | | | | | | |
| --- | --- | --- | --- | --- | --- | --- |
| Tokyo 2020 Olympic | n | Age (SD) | | Women (%) | |  |
| Archery | 10 | 27.3 | (5.9) | 5 | (50.0) |  |
| Athletics | 125 | 25.2 | (3.9) | 40 | (32.0) |  |
| Badminton | 22 | 26.5 | (2.9) | 13 | (59.1) |  |
| Baseball | 26 | 28.1 | (3.5) | 0 | (0.0) |  |
| Basketball | 72 | 26.9 | (4.4) | 38 | (52.8) |  |
| Boxing | 13 | 24.8 | (3.1) | 5 | (38.5) |  |
| Canoe | 16 | 29.3 | (3.5) | 7 | (43.8) |  |
| Clay pigeon Shooting | 7 | 35.9 | (7.7) | 4 | (57.1) |  |
| Cycling | 29 | 28.2 | (5.7) | 13 | (44.8) |  |
| Equestrian | 15 | 39.3 | (7.0) | 2 | (13.3) |  |
| Fencing | 29 | 26.1 | (4.8) | 12 | (41.4) |  |
| Golf | 14 | 25.6 | (4.1) | 8 | (57.1) |  |
| Gymnastics | 49 | 22.4 | (4.4) | 30 | (61.2) |  |
| Handball | 76 | 27.3 | (4.0) | 35 | (46.1) |  |
| Hockey | 56 | 26.6 | (3.2) | 31 | (55.4) |  |
| Judo | 28 | 26.4 | (2.8) | 14 | (50.0) |  |
| Karate | 16 | 28.4 | (3.0) | 8 | (50.0) |  |
| Modern Pentathlon | 8 | 26.9 | (5.9) | 4 | (50.0) |  |
| Rifle shooting | 8 | 31.3 | (6.8) | 4 | (50.0) |  |
| Rowing | 8 | 27.6 | (2.5) | 4 | (50.0) |  |
| Rugby Sevens | 53 | 26.5 | (4.6) | 30 | (56.6) |  |
| Sailing | 13 | 30.9 | (6.0) | 7 | (53.8) |  |
| Skateboarding | 18 | 18.2 | (2.9) | 12 | (66.7) |  |
| Soccer | 56 | 24.3 | (3.5) | 36 | (64.3) |  |
| Softball | 19 | 29.0 | (4.7) | 19 | (100.0) |  |
| Sport Climbing | 4 | 25.8 | (4.4) | 2 | (50.0) |  |
| Surfing | 5 | 21.8 | (2.8) | 3 | (60.0) |  |
| Swimming | 95 | 24.2 | (4.3) | 51 | (53.7) |  |
| Table tennis | 12 | 23.1 | (4.7) | 6 | (50.0) |  |
| Taekwondo | 8 | 22.9 | (4.3) | 4 | (50.0) |  |
| Tennis | 21 | 27.4 | (5.6) | 12 | (57.1) |  |
| Triathlon | 12 | 29.0 | (4.3) | 7 | (58.3) |  |
| Volleyball | 65 | 29.0 | (5.7) | 30 | (46.2) |  |
| Weightlifting | 19 | 28.8 | (2.9) | 10 | (52.6) |  |
| Wrestling | 13 | 26.4 | (3.4) | 6 | (46.2) |  |
| Tokyo 2020 Paralympic |  |  | |  | |  |
| Archery | 8 | 39.5 | (9.0) | 2 | (25.0) |  |
| Athletics | 15 | 32.5 | (10.8) | 3 | (20.0) |  |
| Canoe | 6 | 34.2 | (10.3) | 3 | (50.0) |  |
| Powerlifting | 14 | 43.9 | (9.2) | 3 | (21.4) |  |
| Rowing | 5 | 32.8 | (13.0) | 3 | (60.0) |  |
| Taekwondo | 4 | 30.0 | (3.2) | 1 | (25.0) |  |
| Wheelchair Fencing | 10 | 38.7 | (7.5) | 3 | (30.0) |  |
| Wheelchair Rugby | 16 | 33.6 | (8.4) | 1 | (6.2) |  |
| Wheelchair Tennis | 5 | 33.8 | (5.1) | 1 | (20.0) |  |
| Beijing 2022 Olympic |  |  | |  | |  |
| Bobsleigh・Luge・Skeleton | 7 | 26.7 | (5.7) | 0 | (0.0) |  |
| Curling | 11 | 31.4 | (5.7) | 6 | (54.5) |  |
| Ice hockey | 20 | 26.4 | (4.8) | 20 | (100.0) |  |
| Skating | 72 | 24.5 | (4.5) | 32 | (44.4) |  |
| Skiing | 129 | 23.9 | (5.4) | 52 | (40.3) |  |
| Beijing 2022 Paralympic |  |  | |  | |  |
| Skiing | 28 | 34.4 | (8.7) | 8 | (28.6) |  |
| Wheelchair curling | 2 | 54.5 | (2.1) | 1 | (50.0) |  |

Data are represented as mean (SD) for age and number (%) for gender rate.
