# Supplementary material for: Investigation of supplement use and knowledge among Japanese elite athletes for the Tokyo 2020 Olympic/Paralympic games and the Beijing 2022 winter Olympic/Paralympic games
Source: Front Sports Act Living. 2023 Oct 18;5:1258542. doi: 10.3389/fspor.2023.1258542 (PMC10622793; doi:10.3389/fspor.2023.1258542)
Supplement: Supplementary file 2 [file Datasheet1.docx]

Appendix 1

**【Nutrition】**

- Please answer how many days per week you typically do to the following ①～⑨ activities.
  Additionally, please answer how many servings (SVs) per day you eat of the followings according to the example below. Please refer to “How to calculate how many servings per day you eat” on the next page.

| Question | Frequency |
| --- | --- |
| 1. Having a staple food, main dish, and side dish ≥ twice/day | days/wk |
| 1. Having **all three meals** (breakfast, lunch, and dinner)  in a day | days/wk |
| 1. Having confectionery or soft drinks (sweetened juice, sweetened canned coffee, etc.) *** Excluding sports drinks** | days/wk |
| 1. Drinking alcohol **(Product：　　　　　　　　　　　　　)** | days/wk |

- Please answer either “Yes” or “No” to the following ①～⑤ items:

| 1. Do you know the significance of snacking in sports (function, role, and usage)? | Yes | No |
| --- | --- | --- |
| 1. Do you take sports foods (sports drinks, jelly, gels, blocks, bars, etc.)? | Yes | No |
| 1. Do you hydrate during your practices or competitions? | Yes | No |
| 1. Have you ever had any nutritional support (seminars or counseling)? | Yes | No |
| 1. Do you use any supplements? | Yes | No |

- If you primarily answered “Yes” to (9), please answer the following questions:

Do you carefully pay attention to anti-doping recommendations when using supplements?

□ Yes　　　　　　□ No

9）Based on the supplements you have used in the past year, **please select the most suitable number** from the table below: -① Ingredients (if multiple ingredients included, **please select all**), -② Reasons.

| ① Ingredients | |
| --- | --- |
| 1. Protein powder (whey, casein, soy, pea, etc., [e.g., Amino protein, Junior protein]） | |
| 2. Amino acids (BCAA, glutamine, glycine, arginine, citrulline, etc.,  [e.g., Amino VITAL PRO or GOLD, Glyna]) | |
| 3. Vitamins (vitamin C, vitamin D, vitamin E, etc., [e.g., multi-vitamin]) | |
| 4. Mineral (calcium, iron, zinc, magnesium, potassium, sodium, etc., [e.g., multi-mineral, OXYUP]) | |
| 5. Fatty acids (DHA, EPA, omega-3, MCT, etc., [e.g., fish oil]) | |
| 6. Probiotics (lactobacillus, bifidobacterium, L-92, L-137, etc.) | |
| 7. Creatine | 8. Caffeine (e.g., energy drink) |
| 9. β-alanine | 10. Sodium bicarbonate, sodium citrate |
| 11. HMB | 12. Others |
| ② Reasons | |
| 1. For weight gain or weight loss | |
| 2. For recovery | |
| 3. For performance enhancement | |
| 4. For energy or nutrients supplementation | |
| 5. To treat or prevent disease and injury or strengthen immune system | |
| 6. For improved sleep quality | |
| 7. For improved intestinal environment | |
| 8. Recommended by staff (coach, teammate, someone from the product company, etc.) | |
| 9. Teammates or other players use it | |
| 10. It was free (from product company, team, cafeteria, etc.,) | |
| 11. It is convenient to supplement energy or nutrients before and/or after exercise | |
| 12. Just in case | |
| 13. Others (　　　　　　　　　　　　　　　　　　　　　　　　　　　) | |

10）Before using the supplements, did you get diagnosed by a doctor or assessed by a dietitian?

Yes  No

If you answered “Yes” to the question above, please check the following ①~② items:

| ① -1 The doctor diagnosed undernutrition | | | Yes | | No |  |
| --- | --- | --- | --- | --- | --- | --- |
| ①-2 The dietitian assessed undernutrition using dietary records | | | Yes | | No |  |
| ② Considered whether the targeted nutrients cannot be obtained from dietary foods (due to food allergy or training abroad) | Yes | No | | Not this   purpose | | |
| ③ Confirmed that the supplements improve the targeted nutrient deficiency. | Yes | No | | Not this   purpose | | |
| ④ Confirmed scientific evidence of enhanced performance | Yes | No | | Not this   purpose | | |
| ⑤ Confirmed side effects or interactions between supplements and medications | Yes | No | |  | | |

10)-⑥ Please check all of the information sources that encourage you to use supplements.

| 1. Your own research (internet, book,  advertisement, etc.) | 7. Doctor |
| --- | --- |
| 2. Coach | 8. Pharmacist |
| 3. Trainer | 9. Teammate |
| 4. Salesperson from a product company | 10. Family member |
| 5. Dietitian from a product company | 11. Friend (including seniors or juniors） |
| 6. Dietitian not from a product company | 12. Others (　　　　　　　　　　　) |

11）If you primarily answered “No” to (9) , please check the following (multiple answers available):

| 1. No need |
| --- |
| 2. Unsure if it is effective |
| 3. Worried about doping |
| 4. Experiencing side effects |
| 5. Team staff (head coach, coach, trainer, etc.) do not recommend it |
| 6. Family does not recommend to use it |
| 7. Others（　　　　　　　　　　　　　　　　　　　　　　　　　　　　　　　　　　　　　） |
